# How to Be 80 Year Old and Have a VO_2max_ of a 35 Year Old

**DOI:** 10.1155/2015/909561

**Published:** 2015-02-18

**Authors:** Trine Karlsen, Ingeborg Megård Leinan, Fredrik Hjulstad Bækkerud, Kari Margrethe Lundgren, Atefe Tari, Sigurd Loe Steinshamn, Asbjørn Støylen, Øivind Rognmo

**Affiliations:** ^1^K.G. Jebsen Center of Exercise in Medicine, Department of Circulation and Medical Imaging, Norwegian University of Science and Technology, 7491 Trondheim, Norway; ^2^St. Olav's University Hospital, Trondheim, Norway; ^3^Department of Pulmonary Medicine, St. Olav's University Hospital, Trondheim, Norway; ^4^Department of Cardiology, St. Olav's University Hospital, Trondheim, Norway

## Abstract

*Background*. To discuss the cardiovascular and pulmonary physiology and common risk factors of an 80-year-old man with a world record maximal oxygen uptake of 50 mL·kg^−1^·min^−1^. *Methods*. Case report. *Results*. His maximal oxygen uptake of 3.31 L·min^−1^, maximal heart rate of 175 beats·min^−1^, and maximal oxygen pulse of 19 mL·beats^−1^ are high. He is lean (66.6 kg) and muscular (49% skeletal muscle mass). His echo parameters of mitral flow (left ventricular filling, *E* = 82 cm·s^−1^ and *E/A* = 1.2) were normal for 40- to 60-year-old men. Systolic and diastolic function increased adequately during exercise, with no increase in left ventricular filling pressure. He has excellent pulmonary function (FVC = 4.31 L, FEV1 = 3.41, FEV1/FVC = 0.79, and DLCO = 12.0 Si^1^) and normal FMD and blood volumes (5.8 L). He has a high level of daily activity (10,900 steps·day^−1^ and 2:51 hours·day^−1^ of physical activity) and a lifelong history of physical activity. *Conclusion*. The man is in excellent cardiopulmonary fitness and is highly physically active. His cardiac and pulmonary functions are above expectations for his age, and his VO_2max_ is comparable to that of an inactive 25-year-old and of a normal, active 35-year-old Norwegian man.

## 1. Introduction

A recent study reported world-record maximal oxygen uptake (VO_2max⁡_) for octogenarian endurance athletes of between 34 and 42 mL·kg^−1^·min^−1^, with reference values for inactive subjects being 21 mL·kg^−1^·min^−1^ [1]. It is well known that VO_2max⁡_ reduces with aging [2, 3], and several factors, including systolic and diastolic heart function, pulmonary function and diffusion capacity, peripheral circulation, and skeletal muscle metabolism and mass, may limit VO_2max⁡_ [4–6]. Maintaining a high VO_2max⁡_ may be essential for healthy aging, because VO_2max⁡_ is a strong and independent predictor of mortality [7] and protects against cardiovascular mortality and morbidity [8]. Therefore, understanding how to maintain it is vital. The purposes of this publication are to describe the physiology of an 80-year-old man with an extraordinarily high VO_2max⁡_ of 50 mL·kg^−1^·min^−1^ and to discuss how it is possible to have “VO_2max⁡_ of a 35-year-old” at 80 years of age. The man's impressively high VO_2max⁡_, combined with a lifelong history of physical activity, is unique and could be a world record at his age.

## 2. Methods

The case was investigated in February and March 2013. First, resting blood pressure and heart rate (Phillips IntelliVue MP50, Germany) and body composition by bioimpedance (InBody 720, Biospace Co., Ltd., Seoul, Korea) were measured, followed by a VO_2max⁡_ treadmill-running test (breath-by-breath measurement with the MMX-II, CORTEX Biophysik GmbH, Germany). The test-retest reliability for the equipment used in this study has previously been reported to have a test-retest correlation for oxygen uptake of 0.99, *p* < 0.001, with a coefficient of variation of 1.8% [9]. After a 15-minute warm-up, the VO_2max⁡_ was tested with treadmill running on a fixed inclination of 10% and an increase in speed approximately every minute until exhaustion. VO_2max⁡_ was defined as the mean of the highest three consecutive 10-second measurements where the VO_2_ leveled off despite an increase in speed. The results of the VO_2max⁡_ test were reconfirmed twice in two test laboratories (once in our laboratory and once in a local Olympic training facility laboratory).

Second, after 12 hours of fasting, flow-mediated dilatation (FMD) in the brachial artery was measured with a vascular ultrasound (Vivid 7, GE Vingmed, Norway) [10]; blood samples were collected and analyzed at the St. Olav's hospital's biochemical laboratory, and the total blood, plasma, erythrocyte volume, and hemoglobin mass were measured with the optimized carbon monoxide rebreathing method, where carbon monoxide serves as a marker of hemoglobin (Bayreuth, Germany) [11]. Furthermore, flow-volume spirometry and diffusion capacity were measured with standard procedures (SensorMedics Vmax Encore 22, Homestead, FL, USA), and resting and exercise cycling cardiac echocardiography were performed with an increasing protocol until the heart rate reached ~100 beats·min^−1^ (Vivid 7, GE Vingmed, Norway) with simultaneous electrocardiogram ECG recordings (Siemens Medical, Germany). In addition, the man wore a physical activity sensor for 6 days (Armband, Senseware, USA).

The man volunteered to be investigated and have the results published as a case report. The Central Norway Regional Ethical Committee was consulted for ethical advice; however, no application was needed, because the study did not fall under the Norwegian law for medical research. The authors were advised to write an informed consent to the man, explaining in detail the physiological examinations and the publication plan. The authors complied, and the consent form was signed by the man before the physical examinations.

## 3. Results 

**Figure 1:**
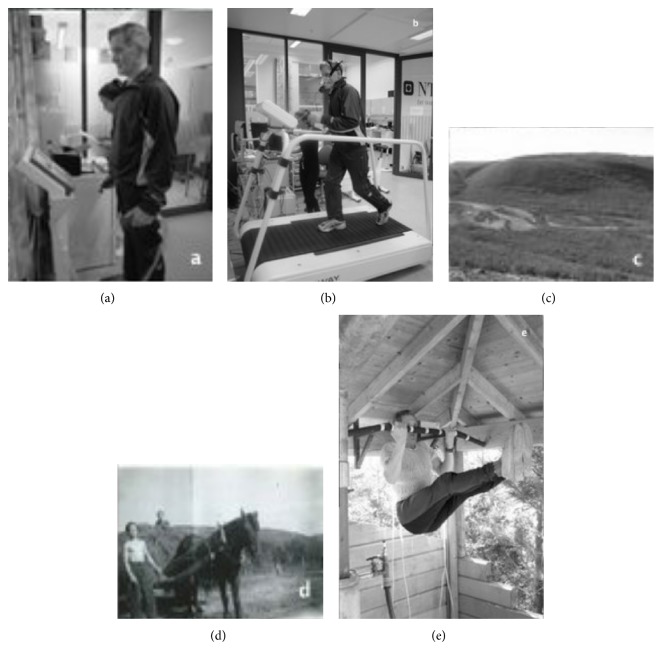
(a) The man measuring body composition. (b) The man running the VO_2max⁡_ test. (c) His childhood farm in Northern Norway. (d) The man doing farm work as a 13-year-old. (e) The man engaged in strength-exercise training in his home gym.

The man is lean and muscular with a strong upright posture while standing (Figure 1(a)). He has never smoked and is currently medicated with aspirin. No definite indication for aspirin could be found according to established guidelines; the reason for the treatment may be due to the pacemaker, combined with the subjects' age, being taken as an indication of general cardiovascular disease. His blood biomarkers are normal, including normal renal function, and his other demographic variables are displayed in Table 1.

His VO_2max⁡_ is 50 mL·kg^−1^·min^−1^, corresponding to 3.31 L·min^−1^ and 14 METs (14 standard metabolic equivalents) (Figure 1(b)). At maximal effort, his ventilation (VE) was 111 L·min^−1^, and his breathing frequency was 41 L·min^−1^. His maximal heart rate was 175 beats·min^−1^, and his oxygen pulse (O_2_-pulse) was 19 mL·beats^−1^. His anaerobic threshold was at 86% of VO_2max⁡_ (VO_2_ = 42.8 mL·kg^−1^·min^−1^). The VE/VCO_2_ slope was 29.7. His maximal running speed was 10 km·hr^−1^ at 10% treadmill inclination, and his maximal RER was 1.14, indicating a high effort during the test.

He received a pacemaker ~10 years ago and is in a pacemaker rhythm at rest. His lowest resting heart rate was 52 beat·min^−1^ during sleep and 60 beats·min^−1^ at daytime. The pacemaker was inserted due to intermittent second-degree AV-block, at the time diagnosed as Mobitz type 2. His cardiac function is described in Table 2, which shows abnormal cardiac motion patterns at rest due to the pacemaker rhythm. During a light-intensity, upright bicycle test, he immediately went into sinus rhythm when he started to pedal, and his heart's motion patterns normalized. Even in pacemaker rhythm, his echo parameters of mitral flow (left ventricular filling) were normal for 40- to 70-year-old men in the HUNT study [12]. There were adequate increases in both systolic and diastolic function during exercise, with no increase in left ventricular filling pressure. Findings were in accordance with a highly trained group of 74-year-old men who were previously studied [4]. His baseline brachial artery diameter was 4.41 mm, and his FMD was 2.72%.

His pulmonary function was above average for his age. His forced ventilator capacity (FVC) was 4.31 L, his forced expiratory volume in one second (FEV1) was 3.41 L, and his FEV1/FVC ratio was 0.79. His lung diffusion capacity was high, with a DLCO of 12.0 Si^1^. Maximal voluntary ventilation (MVV) was calculated using FEV1 × 40 [13]. Estimated MVV was 136 L·min^−1^, and the ratio between VE at maximal exercise and the calculated MVV was 0.82. His breathing reserve was 25 L·min^−1^.

His total blood, plasma, and erythrocyte volumes were 5.8, 3.6, and 2.2 L, respectively, and his total Hb-mass was 744 g, with hemoglobin and hematocrit values of 14.1 g·dL^−1^ and 43%. When expressed per kilogram of body mass, his blood, plasma, and erythrocyte volumes were 87, 54, and 33 mL·kg^−1^, respectively, and his total Hb-mass was 11.2 g·kg^−1^.

He has a lifelong history of endurance and strength-exercise training. Born and raised on a small farm in a roadless mountain region, his childhood was dominated by the vigorous manual labor of farm work, fishing, hunting, and berry harvesting (Figures 1(c) and 1(d)), as well as exercise training. He has continued this lifestyle as an adult. He currently self-reports ~30 minutes of structured endurance and strength training 3 times/week (Figure 1(e)), ~20 minutes of endurance training, including short durations with moderate-to-lactate threshold intensity, and ~ 10 minutes of strength training three times per week, mainly on the upper body and core with 10–12 repetitions per set. Each year, he takes a 7-day ski trip in the mountains and has competed in ultraendurance ski races. His current objectively measured daily activity level is high at 10,843 steps·day^−1^. His total energy expenditure (TEE) was 2,476 Kcal·day^−1^, out of which 877 Kcal·day^−1^ was due to active energy expenditure (AEE). Daily active time (≥3 METs) was 2:51 hr·day^−1^, with 2:31 hr·day^−1^ of moderate activity (3–6 METs) and 0:21 hr·day^−1^ of vigorous activity (6–9 METs), while no time of >9 METs was logged. We are unable to confirm any sports competition results from his early or middle age, but online result services from the two major ultraendurance cross-country ski competitions in Scandinavia, the* Birkebeiner* (54 km) and the* Vasaloppet *(90 km) ski races, show his participation and completion in three races. In 2007, he completed the* Vasaloppet* race and, in 2002 and 2004, he completed the* Birkebeiner* race with finishing times of 4:41:25 and 4:46:04 hours, respectively, coming in as number 130 (65–69-year age group) and number 21 (70–74-year age group) in his respective competition groups. In 2004, he also competed in the* Birkebeiner* mountain bike race (89 km) and the* Birkebeiner* mountain half marathon (21 km). His finishing times were 4:51:52 and 02:01:45 hours, making him number 5 and number 6 in his age class (70–74-year age group).

## 4. Discussion 

The man has a high VO_2max⁡_ for his age, ~50% above the mean for 80-year-old men in Norway [2] and above the 90 percentile (44.2 mL·kg^−1^·min^−1^) in the ACSM guidelines [14]. His VO_2max⁡_ is higher than the mean of 30- to 39-year-old Norwegian men and inactive 20- to 29-year-old Norwegian men [2], and it is also 8 mL·kg^−1^·min^−1^ above the highest VO_2max⁡_ reported among 12 Swedish lifelong octogenarian endurance athletes, including a former Olympic champion [1]. His MET value of 14 is high, which places him in the low mortality risk category ≥10 METs [8, 15] as well as above the 44.2 mL·kg^−1^·min^−1^ threshold where an unfavorable cardiovascular risk profile is apparent in Norwegian men [2]. This is supported by his measured risk parameters. The man reports having been tested twice (at 25 and 45 years of age) with cardiopulmonary exercise as part of his work health services, which resulted in a VO_2max⁡_ of ~75 and ~58 mL·kg^−1^·min^−1^. This represents a relative 7% reduction in VO_2max⁡_ per decade, which is comparable to the 6–11% reduction per decade reported elsewhere in both well-trained and sedentary men [1, 3, 16–19]. His estimated absolute reduction in VO_2max⁡_ of 0.45 mL·kg^−1^·min^−1^ per year from 25 to 80 years of age is highly comparable to values reported for active subjects in the meta-analysis by Wilson and Tanaka (4.6 mL·kg^−1^·min^−1^ per decade) [18] and in endurance-trained men in the cross-sectional study of Pimentel et al. (5.4 mL·kg^−1^·min^−1^ per decade) [19]. However, he reports that the majority of his decrease in VO_2max⁡_ with increasing age occurred in between 25 and 45 years of age, with 8.5 mL·kg^−1^·min^−1^ decrease per decade. Between 45 and 80 years of age, his VO_2max⁡_ decreased by only 0.23 mL·kg^−1^·min^−1^ per year. This is different from what is reported by others, where a minor decrease in VO_2max⁡_ was seen before the age of 50 years and the majority of the VO_2max⁡_ decrease presented beyond this age [19]. The man reports being seriously injured at the age of 42 years, and this could explain the larger degree of decrease in VO_2max⁡_ before the age of 50 years as this injury made him inactive for a period of time. Because the man is highly physically active at the age of 80 and also reports this as being his normal lifestyle, this may explain how he has maintained his absolute VO_2max⁡_ to a greater extent from 50 years of age compared to others, whose reported reduction in VO_2max⁡_ corresponds with a lesser volume of exercise training [19].

His cardiac function (Table 2) is higher than normal for left ventricular volume, with normal function parameters. Despite being in pacemaker rhythm, his diastolic function was normal (tissue Doppler) to super normal (mitral flow), compared to the values from the Norwegian HUNT study [12]. His maximal O_2_ pulse was high with 19 mL·beats^−1^, which is, respectively, 9% and 15% above men his age in Norway [9] and lifelong Swedish athletes [1], indicating a high maximal stroke volume. He has a high maximal heart rate for his age, 12 beats·min^−1^ higher than the mean of Norwegian men his age [9, 20]. Together with a high O_2_ pulse, this indicates a large cardiac output during exercise. His total blood, plasma, and erythrocyte volumes were ~17–19% higher than those in the published data on older men [21]. Moreover, these volumes were at the levels of normal-to-moderate performance endurance athletes [22] and yet below the values of elite athletes [23]. He clearly has an “athlete's” heart with bradycardia; however, no information on this exists from earlier in his life.

His vascular function was normal for his age and gender, with an FMD of 2.7%. His FMD is 3% below the mean of a Norwegian reference population, but he has a large baseline diameter and no indication of endothelial dysfunction, because his FMD is above 0% [24]. His blood biomarkers were also normal and low for his age as was his blood pressure, with no indication of elevated cardiovascular risk markers.

His lung function was above average for his age. His FVC, FEV1, and FEV1% were at 128%, 126%, and 97% of the age-predicted values, respectively. His diffusion capacity was excellent, with a DLCO value at 149% of the age-predicted value. With a maximal ventilation of 111 L·min^−1^, his breathing reserve is normal at 25 L·min^−1^ and 82% of predicted MVV, which is comparable to an age reference value of 26.6 L·min^−1^ [16]. Without his supranormal dynamic lung volumes, he probably would have been ventilatory-limited as a “normal for age” FEV1 of approximately 2.70 L, giving a calculated MVV of 108 L, which is below his maximal VE of 111 L. His VE/VCO_2_ slope is normal, despite his high age, indicating a good match of ventilation and perfusion in the pulmonary ventilation, and he has a maximal VE/VO_2_ of 33.5, indicating a normal ventilatory cost for his oxygen uptake [25].

Despite self-reporting a low volume of systematic exercise training (90 min per week), he is highly physically active, engaging in 83% more physical activity than the minimum daily recommendation [26]. He walks ~4,100 steps·day^−1^ more than the mean of Norwegian men above 65 years old and ~2,650 steps·day^−1^ more than Norwegian men between 20 and 64 years [27]. He outwalks the octogenarian lifelong Swedish athletes by ~2,800 steps·day^−1^ and untrained men by ~6,500 steps·day^−1^. He is moderately and vigorously active, 1050 min·wk^−1^ and 147 min·wk^−1^, which is seven times and two times above the recommendations for moderate and high-intensity physical activity of ≥150 min·wk^−1^ and ≥75 min·wk^−1^, respectively [26]. His habits may indicate that large volumes of both moderate and vigorous physical activity are needed to maintain a high VO_2max⁡_ at this age, in contradiction to what has been reported by others [18]. The man describes the week he wore the activity sensor as a normal week for him in terms of physical activity. As the sports competition results we have are limited to cross-country ski races when the man was 69, 71, and 74 years of age, we are unable to discuss the change in performance from young age. There was a slight increase in his competition time from 2002 to 2004 of 4:39 minutes; however, as the weather, snow, and ski conditions are vital for performance in ski races, we are unable to pinpoint the reason for the ~2% increase in race time. In 2004, he was number 21 in his age group (70–74 years) in the race, while he was number 130 in his age group in 2002 (65–69). Compared to the winning times in his age classes, his race time was a respective 27% and 10% slower than the winner in his age group in 2002 (65–69 years) and 2004 (70–74 years), and this is most likely due to him being on the younger end in his competition group in 2004, while he was oldest within his group in 2002.

In addition to endurance exercise training, he reports performing systematic strength training. His maximal or functional muscle strength was not measured in this study; however, his large muscle mass and ability to perform repeatedly high-demand strength exercises (Figure 1(e)) suggest that a strong and functional skeletal muscle mass may positively affect his high cardiopulmonary fitness.

## 5. Conclusion

The 80-year-old man has excellent “35-year-old” cardiopulmonary fitness with a low cardiovascular risk profile and might have a world-record VO_2max⁡_ for his age and gender due to excellent cardiac function (both systolic and diastolic), pulmonary function, and a good match between the cardiac and pulmonary system. In addition, a large, strong skeletal muscle mass; age-elevated blood volume and hemoglobin mass; and normal arterial vascular endothelial function may contribute positively. He is highly physically active and has a lifelong history of physical activity and exercise training. His highly physically active lifestyle may be his “fountain of youth” at 80 years of age, together with a high level of VO_2max⁡_ at young age.

## Perspectives

It is possible to be an 80-year-old man and have a VO_2max⁡_ of a 35-year-old with a low cardiovascular risk profile. The volume of physical activity for an active and highly fit 80-year-old man is high, seven and two times higher than recommendations for moderate and vigorous physical activity. The reduction in VO_2max⁡_ with aging is normal in the 80-year-old man, indicating the importance of having a high maximal oxygen uptake at a young age.

## Figures and Tables

**Table 1:** Demographic variables.

Body weight (kg)	66.6
BMI	23
Fat mass (%)	12
Skeletal muscle mass (%)	49
Waist-to-hip ratio	0.85
Systolic blood pressure (mmHg)	136
Diastolic blood pressure (mmHg)	78
Total cholesterol (mmol·L^−1^)	4.00
HDL cholesterol (mmol·L^−1^)	1.76
LDL cholesterol (mmol·L^−1^)	1.99
Triglyceride (mmol·L^−1^)	0.55
TSH (mIE·L^−1^)	1.75
Blood glucose (mmol·L^−1^)	5.2
B-HbA1c (%)	6.2
C-peptide (nmol·L^−1^)	0.3
Creatinin (*μ*mol·L^−1^)	82
Estimated GRF (mL·min^−1^)	78

Demographic description of the case. BMI: body mass index, GRF: glomerular filtration rate, HDL: high density lipoproteins, LDL: low density lipoproteins, TSH: thyroid stimulating hormone.

**Table 2:** Cardiac findings at rest and light exercise with echocardiography.

Rest	
Heart rate	60
Rhythm	Pacemaker
End diastolic volume (mL)	147
Ejection fraction (%)	54
Mitral annulus displacement (cm)	1.2
Mitral flow *E* (cm·s^−1^)	82^*^
Mitral flow *E*/*A*	1.2^*^
*E* Deceleration time (ms)	199^*^
Isovolumic relaxation time (ms)	94^*^
Tissue Doppler systolic velocity (*S*′—cm·s^−1^)	8.0^§^
Tissue Doppler early diastolic velocity (*e*′—cm·s^−1^)	7.5^§^
*E*/*e*′	10.9^§^

Light exercise	
Heart rate	100
Rhythm	Sinus
End diastolic volume (mL)	124
Ejection fraction (%)	80
Mitral flow *E* (cm·s^−1^)	112
Tissue Doppler early diastolic velocity (*e*′—cm·s^−1^)	12.0
*E*/*e*′	10.0

Echocardiographic findings at rest and during light, upright bicycle exercise. The ∗ denotes normal values for males in the age interval of 40–60 years in the HUNT study, while § denotes values that are normal for males in the age range of >60 [12].

## References

[1] Trappe S., Hayes E., Galpin A. (2013). New records in aerobic power among octogenarian lifelong endurance athletes. *Journal of Applied Physiology*.

[2] Aspenes S. T., Nilsen T. I. L., Skaug E. A. (2011). Peak oxygen uptake and cardiovascular risk factors in 4631 healthy women and men. *Medicine and Science in Sports and Exercise*.

[3] Trappe S. W., Costill D. L., Vukovich M. D., Jones J., Melham T. (1996). Aging among elite distance runners: a 22-yr longitudinal study. *Journal of Applied Physiology*.

[4] Molmen H. E., Wisloff U., Aamot I. L., Stoylen A., Ingul C. B. (2012). Aerobic interval training compensates age related decline in cardiac function. *Scandinavian Cardiovascular Journal*.

[5] Richardson R. S., Harms C. A., Hepple R. T. (2000). Skeletal muscle: master or slave of the cardiovascular system?. *Medicine and Science in Sports and Exercise*.

[6] Bassett D. R., Howley E. T. (2000). Limiting factors for maximum oxygen uptake and determinants of endurance performance. *Medicine and Science in Sports and Exercise*.

[7] Myers J., Prakash M., Froelicher V., Do D., Partington S., Atwood J. E. (2002). Exercise capacity and mortality among men referred for exercise testing. *The New England Journal of Medicine*.

[8] Blair S. N., Kohl H. W., Paffenbarger R. S., Clark D. G., Cooper K. H., Gibbons L. W. (1989). Physical fitness and all-cause mortality: a prospective study of healthy men and women. *Journal of the American Medical Association*.

[9] Loe H., Rognmo Ø., Saltin B., Wisløff U. (2013). Aerobic capacity reference data in 3816 healthy men and women 20–90 years. *PLoS ONE*.

[10] Corretti M. C., Anderson T. J., Benjamin E. J. (2002). Guidelines for the ultrasound assessment of endothelial-dependent flow-mediated vasodilation of the brachial artery: a report of the international brachial artery reactivity task force. *Journal of the American College of Cardiology*.

[11] Schmidt W., Prommer N. (2005). The optimised CO-rebreathing method: a new tool to determine total haemoglobin mass routinely. *European Journal of Applied Physiology*.

[12] Dalen H., Thorstensen A., Vatten L. J., Aase S. A., Stoylen A. (2010). Reference values and distribution of conventional echocardiographic Doppler measures and longitudinal tissue Doppler velocities in a population free from cardiovascular disease. *Circulation: Cardiovascular Imaging*.

[13] Campbell S. C. (1982). A comparison of the maximum voluntary ventilation with the forced expiratory volume in one second: an assessment of subject cooperation. *Journal of Occupational Medicine*.

[14] Whaley M. H. (2006). *ACSM’s Guidelines for Exercise Testing and Perscription*.

[15] Kokkinos P., Myers J. (2010). Exercise and physical activity: clinical outcomes and applications. *Circulation*.

[16] Edvardsen E., Hansen B. H., Holme I. M., Dyrstad S. M., Anderssen S. A. (2013). Reference values for cardiorespiratory response and fitness on the treadmill in a 20- to 85-year-old population. *Chest*.

[17] Heath G. W., Hagberg J. M., Ehsani A. A., Holloszy J. O. (1981). A physiological comparison of young and older endurance athletes. *Journal of Applied Physiology Respiratory Environmental and Exercise Physiology*.

[18] Wilson T. M., Tanaka H. (2000). Meta-analysis of the age-associated decline in maximal aerobic capacity in men: relation to training status. *The American Journal of Physiology—Heart and Circulatory Physiology*.

[19] Pimentel A. E., Gentile C. L., Tanaka H., Seals D. R., Gates P. E. (2003). Greater rate of decline in maximal aerobic capacity with age in endurance-trained than in sedentary men. *Journal of Applied Physiology*.

[20] Nes B. M., Janszky I., Wisløff U., Støylen A., Karlsen T. (2013). Age-predicted maximal heart rate in healthy subjects: the HUNT Fitness Study. *Scandinavian Journal of Medicine and Science in Sports*.

[21] Davy K. P., Seals D. R. (1994). Total blood volume in healthy young and older men. *Journal of Applied Physiology*.

[22] Schmidt W., Prommer N. (2008). Effects of various training modalities on blood volume. *Scandinavian Journal of Medicine & Science in Sports*.

[23] Heinicke K., Wolfarth B., Winchenbach P. (2001). Blood volume and hemoglobin mass in elite athletes of different disciplines. *International Journal of Sports Medicine*.

[24] Skaug E.-A., Aspenes S. T., Oldervoll L. (2013). Age and gender differences of endothelial function in 4739 healthy adults: the HUNT3 fitness study. *European Journal of Preventive Cardiology*.

[25] Guazzi M., Adams V., Conraads V. (2012). EACPR/AHA joint scientific statement. Clinical recommendations for cardiopulmonary exercise testing data assessment in specific patient populations. *European Heart Journal*.

[26] Garber C. E., Blissmer B., Deschenes M. R. (2011). Quantity and quality of exercise for developing and maintaining cardiorespiratory, musculoskeletal, and neuromotor fitness in apparently healthy adults: guidance for prescribing exercise. *Medicine and Science in Sports and Exercise*.

[27] Hansen B. H., Kolle E., Dyrstad S. M., Holme I., Anderssen S. A. (2012). Accelerometer-determined physical activity in adults and older people. *Medicine & Science in Sports & Exercise*.

